# COVID-19: Protocol and Checklist for Nursing Care Management at Urgent Care Units

**DOI:** 10.3390/ijerph20032169

**Published:** 2023-01-25

**Authors:** Leticia Pereira Orestes, Silmara Meneguin, Aniele de Leo, Mayara Salles Gasparini Patini, Bruna Pegorer Santos, Cesar de Oliveira

**Affiliations:** 1 Department of Nursing, Botucatu Medical School, Paulista State University, Botucatu 18618-687, SP, Brazil; 2 Department of Epidemiology & Public Health, University College London, London WC1E 6BT, UK

**Keywords:** coronavirus infections, nursing care, nursing, emergency medical services, validation study

## Abstract

Background: The 24 h urgent care units (24 h UCU) in Brazil are the main pre-admission hospital process of the public healthcare system and constitute an intermediate modality between primary care and hospital care. These units also provide care in cases of less severity that are not considered urgent. This study aimed to create and validate the content of a graphic protocol and checklist for the nursing care management of patients with a suspicion or confirmation of infection by COVID-19 at urgent care units. Methods: A methodological study was carried out in three phases: construct of items and dimensions of the checklist; evaluation of the checklist by specialists for content validation; and construct and content validation of the graphic protocol. Results: The checklist was evaluated by nine specialists. Eight items received suggestions for changes. Items with a content validity index ≥0.83 were maintained. With regard to content validity, despite the satisfactory level of agreement, the specialists suggested some changes in the writing of eight items. The graphic protocol was evaluated by six specialists and had an overall content validity of 0.97. Conclusion: The checklist with 44 items and three dimensions (Management, Biosafety and Care) and the protocol achieved a satisfactory standard of content validity for use at 24 h urgent care units. This protocol can contribute to the standardization and guidance of nursing actions in suspected and confirmed cases of COVID-19 at urgent care units, ensuring safe care based on scientific evidence.

## 1. Introduction

The pandemic caused by SARS-CoV-2, a new virus discovered at the end of 2019, has spread rapidly throughout the world [1]. Brazil occupies third place among countries with the largest number of cases recorded in the world, and second in the number of deaths due to COVID-19 [2].

In mild cases, the clinical manifestation of this disease is characterized by symptoms of the common cold with or without fever and other symptoms. Moderate, severe and critical cases may involve pneumonia and severe acute respiratory syndrome (SARS), with the possibility of sepsis and death [3]. According to the World Health Organization, most people infected by SARS-CoV-2 develop mild (40%) or moderate (40%) forms of the diseases, approximately 15% develop the severe form, and only 5% develop the critical form, which requires intensive care [4]. In a scenario of rapid dissemination, the severity of cases, and difficulties in containing the disease [1], the excessive load on healthcare services has been unavoidable, especially at urgent and emergency care units.

In Brazil, 24 h urgent care units (24 h UCU) are the main pre-admission hospital process of the public healthcare system and constitute an intermediate modality between primary care and hospital care. These units also provide care in cases of less severity that are not considered urgent [5,6]. UCUs offer qualified, resolutive care for acute and chronic conditions, first aid in surgical and trauma cases, and the maintenance of patients under clinical observation for up to 24 h for diagnostic clarification or clinical stabilization. These units also serve as the “rear-guard” for the mobile urgent care service and primary care services [6].

However, the routine of 24 h UCUs, which is to provide initial care for patients, was substantially altered during the pandemic. Despite all measures taken by the tertiary sector to absorb the demand for hospitalization, resources quickly became saturated and patients with moderate to severe forms of COVID-19 often remained at UCUs for several days awaiting a hospital bed, which, in many cases, aggravated the existing problem [7].

With the intention to control the spread of COVID-19 in the country, the Brazilian Health Ministry established flowcharts for services that compose the network of the public healthcare system. One of these flowcharts was specifically for 24 h UCUs, with recommendations for mild, moderate and severe cases to develop joint, organized, comprehensive care throughout the entire network of the system [8,9].

Although the aim of the care guidelines is the organization of the practice of providing care at 24 h UCUs, these guidelines were directed at the entire multidisciplinary team involved in providing care for patients with COVID-19 rather than only the nursing staff. However, as 24 h UCUs have different characteristics across the country in terms of physical space, work process/organization, size of the nursing staff and available equipment, it was difficult to implement these protocols precisely as established [10].

In this new scenario affecting the needs of the staff and the demand for care, nurses at 24 h UCUs needed to develop actions to reorganize work processes and manage care for patients with a suspicion or confirmation of infection by COVID-19 [11]. Nursing care management is a professional exercise that is part of the science of providing care through planning and organization to provide care in a timely, safe, comprehensive manner, ensure the continuity of care, and provide support to the strategic orientations of the services based on the best scientific evidence [12].

Therefore, the aim of the present study was to create and validate a graphic protocol and checklist for nursing care management directed at patients with a suspicion or confirmation of infection by COVID-19 at urgent care units.

## 2. Methods

### 2.1. Study Design and Setting 

A methodological study was conducted at a 24 h UCU in a municipal public healthcare system in the state of São Paulo, Brazil, in the period between April and December 2021. The study was developed in three phases: construct of items and dimensions of the checklist; evaluation of the checklist by specialists for content validation; and construct and content validation of the graphic protocol.

Step 1—Selection of items for the checklist

A comprehensive literature review was carried out using the following databases: Latin American and Caribbean Health Sciences Literature (LILACS), PubMed (National Library of Medicine and National Institutes of Health), Cumulative Index to Nursing and Allied Health Literature (CINAHL), Web of Science (WOS), EMBASE and Scopus.

The search strategy included articles that addressed the following research question: What are the recommendations (clinical practice guidelines and protocols) available in the scientific literature at national and international levels towards the nursing care of COVID-19 patients at urgent care units? The following search terms were selected from the Health Sciences Descriptors list (DeCS/MeSH): coronavirus infection, nursing care, medical emergency services, nursing, clinical protocols and clinical practice guidelines, including their respective synonyms and combined with the Boolean operators OR and AND. 

Therefore, the search strategy used was: (“Coronavirus Infections” OR “Infection, Coronavirus” OR “Infections, Coronavirus” OR “Middle East Respiratory Syndrome” OR “MERS Middle East Respiratory Syndrome” OR “COVID-19”) AND (“Emergency Medical Services” OR “Emergency Services, Medical” OR “Emergency Service, Medical” OR “Medical Emergency Service” OR “Medical Emergency Services” OR “Service, Medical Emergency” OR “Services, Medical Emergency” OR “Medical Services, Emergency” OR “Emergency Medical Service” OR “Medical Service, Emergency” OR “Service, Emergency Medical” OR “Services, Emergency Medical” OR “Prehospital Emergency Care” OR “Emergency Care, Prehospital” OR “Emergicenters” OR “Emergicenter” OR “Emergency Care” OR “Emergency Health Services” OR “Emergency Health Service” OR “Health Service, Emergency” OR “Health Services, Emergency” OR “Service, Emergency Health” OR “Services, Emergency Health” OR “Nursing Care” OR “Care, Nursing” OR “Management, Nursing Care” OR “Nursing Care Management” OR Nursing OR Nursings) AND (“Clinical Protocols” OR “Clinical Protocol” OR “Clinical Research Protocol” OR “Clinical Research Protocols” OR “Protocol, Clinical” OR “Protocol, Clinical Research” OR “Protocols, Clinical” OR “Protocols, Clinical Research” OR “Protocols, Treatment” OR “Research Protocol, Clinical” OR “Research Protocols, Clinical” OR “Treatment Protocol” OR “Treatment Protocols” OR “Practice Guideline” OR “Clinical Practice Guideline” OR “Clinical Guidelines”). 

The inclusion criteria included the original articles addressing the research question, available in their entirety, and published in the last five years in English, Portuguese, and Spanish. Duplicate articles, only abstracts, letters to editors and those that did not address the main research question were excluded. 

**Figure 1:**
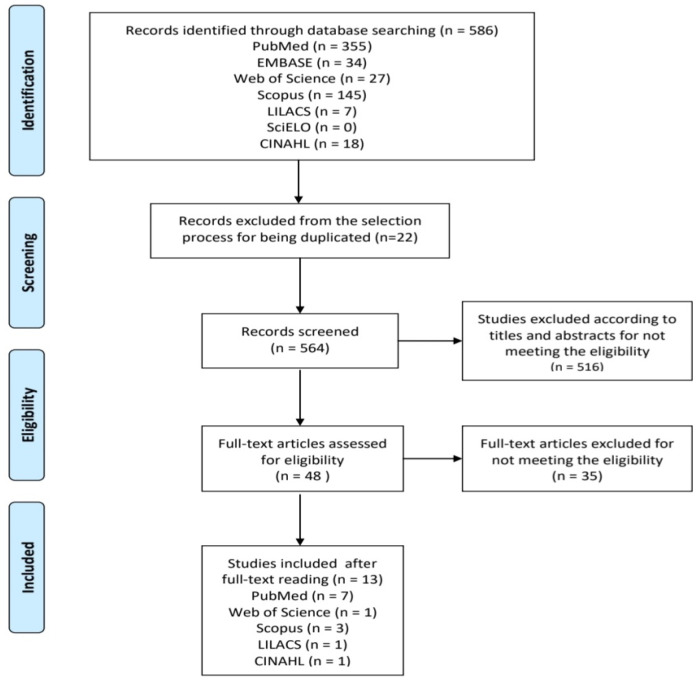
Prisma Flowchart, identification, screening, eligibility and inclusion of studies.

The review was carried out between February and April 2021; 586 articles were found. Then, the authors assessed their titles and abstracts, and duplicates and those articles that did not fulfil the inclusion criteria were excluded. Out of the 48 selected articles, a further 35 were excluded and 13 were eligible to be included in the present study. All these steps have been described in Figure 1. 

Out of the 13 selected articles, only five specifically addressed the aspects of nursing care. The others referred to recommendations for the treatment of cases of COVID-19 at emergency services, particularly cases of cardiorespiratory arrest, airway management, ventilatory support, as well as orientations and strategies for the organization of emergency services.

An additional search was made on the sites of governmental and non-governmental agencies, such as the World Health Organization, Centers for Disease Control and Prevention (CDC), the Brazilian Health Ministry and the National Health Surveillance Agency (ANVISA), for protocols, guidelines, norms, as well as national and international publications. From the bibliographic survey, 13 open-ended questions were created to obtain knowledge on the work dynamics and care provided for patients with COVID-19 at the service, the use of personal protective equipment (PPE) made available by the service and the sequence of actions for putting on and removing garments.

These questions were then sent via e-mail or delivered randomly in print form to 8 nurses and 12 nursing technicians who worked at the institution where the study was conducted due to the impossibility of forming a focus group during the pandemic. The inclusion criteria were being a nursing staff member and working at a 24 h UCU for more than one year. Nurses not currently working, those on vacation and those on medical leave were excluded.

Twelve questionnaires were returned fully answered. The data from these 12 questionnaires were transcribed and organized on a spreadsheet of the Excel^®^ 2019. Next, the data were analysed and interpreted using content analysis proposed by Bardin [13] in three steps: pre-analysis, exploration of the material/treatment of the results and interpretation. A matrix was created with the categories and subcategories identified (Table 1), which gave rise to the dimensions and items of the checklist. 

The theoretical reference was the concept of nursing care management, which consists of the link between the management and care dimensions in the work process of nurses. The actions of nurses involve the organization of the work and human resources in the management dimension and actions focused on integral nursing in the care dimension [12]. For the methodological reference, the literature was used for the development of scales [14] and the Cosmin guidelines were used for content validation [15].

The Management, Biosafety and Care categories were defined as the dimensions of the protocol. Next, items were created addressing nursing actions and care related to each step of the care process.

Step 2—Content validation of the checklist items

Ten nurses, specialists in emergency care or with experience in emergency services and/or researchers with experience in the creation of assessment tools in the health field, with a minimum score of five points using the adapted Fehring criteria were selected considering the following [16]: Doctoral or Master’s degree, with dissertations or theses relevant to the subject of interest, study published on the subject of the interest and at least one year of clinical experience. Participant selection was performed using the “snowball” sampling method. Nurses specialized in emergency care who worked in the region were first invited to compose the group of judges and the researcher then consulted their curricula vitae on the Lattes platform to ensure that these nurses had the minimum score according to the criteria established for the study.

The initial invitation was formal through a letter sent by e-mail or a text message to a cell phone. Each item on the instrument was assessed for clarity, relevance, pertinence and comprehensiveness using a Likert scale ranging from 1 to 4 points [17]. Nine of the nurses agreed to participate in the study. There was only one round of evaluation.

Step 3—Creation and validation of the graphic protocol

Lastly, the protocol with the representation of the entire care process was created in a graphic format as a flowchart to facilitate quick understanding through an overview. The content of the protocol was distributed in accordance with the periods of care (dimensions and subdimensions of care) with the respective items covered in each group. The graphic form of the protocol was created based on that proposed by Pimenta et al. [18] with the use of previously conventionalized, internationally standardized symbols. For validation, the nine specialists who had participated in the content validation of the checklist were invited and used the following criteria: graphic presentation, reading ease, sequence of algorithm, clarity, relevance, pertinence, and comprehensiveness using a Likert scale ranging from 1 to 4 points.

### 2.2. Data Analysis

The content validity index (CVI) was calculated by the sum of agreement on the items scored “3” or “4” in the assessment of the judges divided by the total number of answers in relation to each criterion [17,19,20]. The acceptable agreement rate among the judges for the verification of the validity of the items was established as >0.80 considering the number of judges, as recommended in the literature [21]. Other data were analysed using descriptive statistics. 

### 2.3. Ethical Aspects

This study received authorization of the Research Ethics Committee, protocol n° 4.226.538. The participants signed two copies of the statement of informed consent—one for the participant and the other for the researcher.

## 3. Results

The checklist with 44 items distributed among three dimensions, that is, management, biosafety and care was evaluated by nine specialists, all of whom were women. The mean age was 42 ± 7.5 years and mean time since completing the university education was 17.8 ± 7.4 years.

With regard to content validity, despite the satisfactory level of agreement, the specialists suggested some changes in the writing of eight items, as shown in Table 2. 

With regard to the dimensions, only Item 4 received the suggestion to be allocated to the Management dimension. Items 15, 21 and 44 were received an evaluation of non-compatibility with the corresponding dimension, but the judges did not suggest where these items should be allocated. Thus, among the 44 items on the instrument, 37 remained concentrated in the Care dimension, four in the Management dimension and three in the Biosafety dimension.

The content validity indices and classification of the indices in relation to the items that compose the three dimensions are presented in Table 3.

With regard to the scores attributed by the judges, Item 25 had a score lower than 1 for the criteria of relevance and pertinence and Item 30 had a score lower than 1 for clarity. As the judges made no suggestions for changes to these items and the CVI was 0.88, both items were maintained. Most items received the maximum score in the assessment (CVI = 1.0), which was unanimous for the Comprehensiveness criterion. The CVI was satisfactory on all items for the criteria assessed (CVI ≥ 0.83) and the overall CVI was 0.99.

Thus, the final version of the checklist was composed of 44 items distributed among three dimensions (Management, Biosafety and Care), as listed in Table 4.

**Figure 2:**
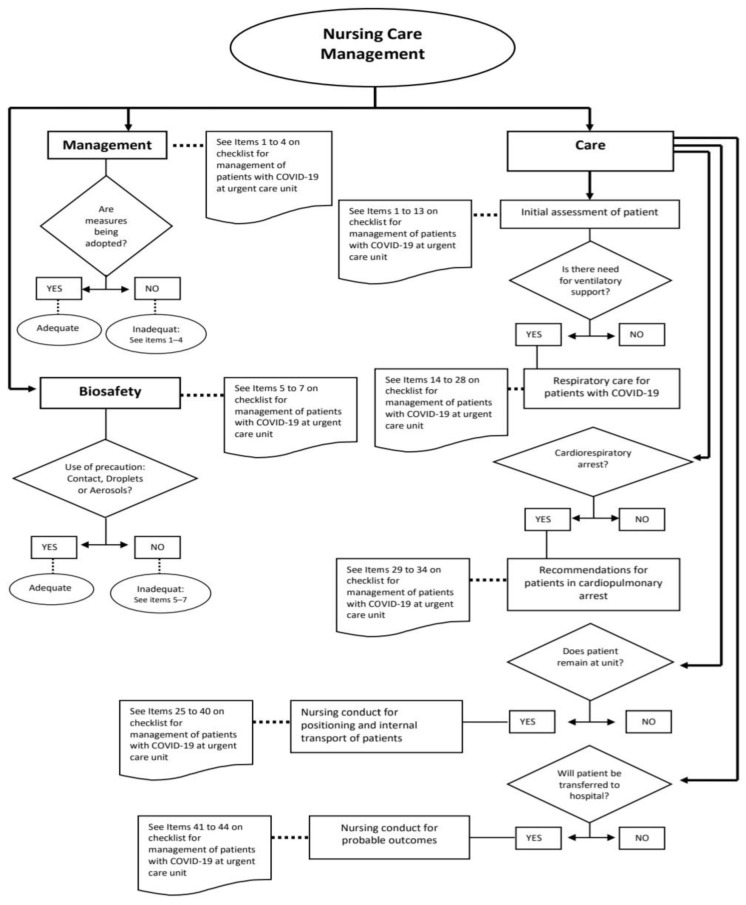
Graphic protocol for nursing care management for patients with suspected or confirmed infection by COVID-19 at a 24 h UCU.

A graphic version of the protocol entitled “Nursing care management for patients with suspected or confirmed COVID-19 at an urgent care unit” was then created using the items on the checklist as reference (Figure 2). The graphic protocol was assessed by six specialists who had participated in the content validation process of the checklist and agreed to participate in this step. All specialists were women, with a mean age of 41.3 ± 3.8 years and a mean of 17.3 ± 2.4 years since the obtainment of the university degree. The overall CVI was 0.97.

## 4. Discussion

The creation and validation of a protocol for the management of patients with a suspicion or confirmation of infection by COVID-19 at 24 h UCUs was developed with methodological and scientific rigor so that it can be used by all nurses despite the diversity of scenarios found at these services across Brazil. Content validation studies are used in nursing research to ensure not only the quality of the instruments created, but also their legitimacy and credibility [22].

A highly satisfactory level of agreement was found among the judges in the assessment of the items and few suggestions were made for changes in the writing of the items. In the assessment of the correspondence of the items to the dimensions in which they were allocated, Item 4 (maintenance of a small team during intubation) received the suggestion to compose the management dimension as the specialist understood this dimension to be linked to the organization of the teams established for the procedure. 

For Items 2 and 3, which address conduct for minimizing the circulation of people and distancing between patients within the service, two judges pointed out problems that could hinder the implementation of the items. One was the unavailability of the radiology service in certain periods, which causes the gathering of patients during long waits, along with the lack of physical space and overcrowding. 

The unavailability of 24 h radiology services at a UCU may be attributed to the lack of human resources. Thus, the solution that the administrators found was to reduce the operating times of this service, as all units have the physical structure for such exams. 

One of the factors that contribute to overcrowding at 24 h UCUs beyond their capacity is related to the perception on the part of the population that access to these services is easier and immediate resources are more ensured, such as appointments, exams and the administration of medications [23]. Another factor that results in overcrowding, and distorts the purpose of 24 h UCUs, is the frequent stay of patients beyond the maximum 24 h of observation established by law as these patients await the availability of a bed in a hospital [7,24].

In the assessment of Item 11, which corresponds to the types of exams with the respective indications for collection for the diagnosis of COVID-19, one specialist commented that the rapid IgM and IgG test is not recommended for the diagnosis of the disease in any patient regardless of the vaccinal situation, serving only on the epidemiological level. In Brazil, the Health Ministry through the Secretary of Health Surveillance establishes the following tests for the laboratory confirmation of cases of COVID-19: molecular biology tests using RT-PCR/RT-LAMP, antigen tests using immunochromatography and the immunological IgM, IgA and/or IgG test using ELISA, immunochromatography (rapid test) for the detection of antibodies, electrochemiluminescence or chemiluminescence immunoassay. IgG results are only used for the confirmation of the disease in non-vaccinated individuals with no previous diagnosis of COVID-19 and with compatible signs and symptoms at least eight days prior to the test [3].

The recommendation of the federal and state epidemiological surveillance agencies is for the IgM/IgGtest not to be used for the diagnosis of COVID-19 in vaccinated individuals due to the expected vaccinal response (production of antibodies) [3]. The CDC [25] recommends the use of antibody tests (serology) for the detection of previous infection by COVID-19 as well as the diagnosis of multisystem inflammatory syndrome in children (MIS-C) and adults (MIS-A) [26], but not for the diagnosis of current infection. In the USA, antibody tests are used for public health surveillance and epidemiological purposes. In the present study, the current national recommendation was used for the creation of the item on the diagnostic confirmation of COVID-19 [3].

Although the experts made no suggestions for changes to the items addressing the placement and removal of PPE, difficulties were encountered in constructing these items due to the divergences found in both the literature and the routine described by the professionals. However, the recommendation used was that proposed by the National Health Surveillance Agency [27] for the placement and removal of PPE when providing care in suspected or confirmed cases of COVID-19. The international literature does not establish a norm for the sequence of putting on hospital garments, and the World Health Organization [28] only mentions the items that are part of individual precaution. The CDC [29] considers more than one safe manner to put on garments, but all are unanimous regarding the recommendation to use a mask, apron, proactive eyewear or face shield, cap and gloves.

Regarding the validation of the graphic protocol, one judge questioned the flow in the care dimension for situations in which the patient does not require ventilatory support or is not in cardiorespiratory arrest. It was not possible to heed this suggestion, as the protocol offers an overall summary of the content, the actions of which are directed by the checklist. Moreover, not all patients require all actions described on the instrument.

The contribution of this study is a nursing management protocol for suspected or confirmed cases of COVID-19 at urgent care units with safety and quality and based on scientific evidence. This protocol could also assist in the creation of similar protocols for other healthcare services, providing further instruments for nursing in the continual quest of improvements in care. 

## 5. Conclusions

The content validation of the protocol checklist was achieved with a satisfactory level of agreement. This agreement was for the individual assessment of the items and overall instrument in accordance with the requirements established in the literature. The same level of agreement was reached among the specialists in the validation of the summarized graphic version of the protocol, that is, the flowchart. 

This protocol can contribute to the standardization and guidance of nursing actions in suspected and confirmed cases of COVID-19 at urgent care units, ensuring safe care based on scientific evidence.

## 6. Recommendations 

Despite its items’ construction being based on a literature review, the instrument used in the present study needs to be reviewed periodically, as new COVID-19 recommendations may be implemented due to new coronavirus variants. Further research should test the proposed checklist to assess its clinical applicability and further improvements. This was an initial study of content validation of an instrument that does not have metric properties. 

## Figures and Tables

**Table 1:** Matrix of categories and subcategories identified in the discourses.

Categories	Definition	Subcategories
Management	Involves actions related to the organization of the service for providing patient care and workflow strategies.	-Differentiated flow for respiratory cases. -Material resources and equipment necessary for providing care in severe cases. -Safety measures recommended. -Strategy developed at the service for the identification of respiratory cases and standardized classification of risk to facilitate recognition on the electronic patient records and prioritize care.
Biosafety	Actions for the prevention, control, reduction, or elimination of the risks inherent to activities that can compromise the health of professionals and patients.	-Measures of professional good practices (use of PPE, protocol for putting on and removing garments).
Care	Actions inherent to direct care by the nursing staff for patients with a suspicion or confirmation of COVID-19.	-Nursing conduct at initial care for patients with suspicion or confirmation of COVID-19. -Criteria for definition, reporting and conduct in cases of severe acute respiratory syndrome—SARS. -Recommendation for ventilatory care: invasive and non-invasive. -Recommendations for providing care in cases of cardiorespiratory arrest. -Nursing conduct regarding positioning of patient and internal transport of patients. -Conduct for transference of patient to reference hospital. -Nursing conduct after death of patient. -Other nursing care not exclusive to cases of COVID-19.

**Table 2:** Summary of suggestions made for times on instrument after evaluation of group of specialists.

Dimension	Suggestion	Item after Change
Management	**Item 1:** Exclude the word “crossing”. **Item 2:** Include examples of common areas and exclude the word “performance” at the end of the sentence.	**Item 1**- Promoted distancing between patients and minimized circulation of people and patients with respiratory symptoms. **Item 2**- Prioritized radiological exam so that patient does not wait in common areas (reception, waiting room, corridor).
Care	**Item 10:** Include “SARS criteria in patients with influenza syndrome”. **Item 12:** Include field for annotating collected answers. **Item 20:** Write HEPA and HMEF out in full. **Item 30:** Explain reason for avoiding the procedure. **Item 34:** Specify side: right or left. **Item 41:** Specify whether transfers to tertiary sector require regulation via CROSS (service regulation center).	**Item 10**- Assessed criteria for severe acute respiratory syndrome (SARS) in patients with influenza syndrome:( ) Dyspnoea OR ( ) Respiratory distress OR ( ) Persistent pressure in chest OR ( ) SpO_2_< 95% in ambient air OR ( ) Cyanosis in lips or face **Item 12**- Investigated patient’s vaccinal situation for COVID-19: Laboratory/Manufacturer of vaccine; Number of doses received **Item 20**- HEPA (High Efficiency Particulate Arrestance) or HMEF (Heat and Moisture Exchanger) filter coupled to orotracheal tube and corrugated extension **Item 30**-Not used manual resuscitator due to high risk of generation of aerosols and contamination of the team. **Item 34**- For defibrillation of patient in PRONE position, sternal paddle was placed in dorsal region and apical paddle was placed on patient’s left side. **Item 41**- Made telephone contact with dispatcher of mobile urgent care service to request transport of patient to hospital reference units after confirmation of availability by CROSS (service regulation center); patient’s current clinical condition as well as the type of transport requested were informed.

**Table 3:** Content validity indices of items in relation to criteria analysed and dimensions proposed by judges. Bauru, São Paulo, 2021.

Item/Dimension 1: Management	C	R	P	Cp
Item 1- Promoted distancing between patients and minimized circulation [...]	1.00	1.00	1.00	1.00
Item 2- Prioritized radiographic exam [...]	1.00	1.00	1.00	1.00
Item 3- Minimized patient’s contact with other patients during stay [...]	1.00	1.00	1.00	1.00
Item 4- Maintained small team, at most, three professionals during intubation [...]	1.00	1.00	1.00	1.00
**Item/Dimension2: Biosafety**				
Item 5- Put on garments prior to onset of patient care [...]	1.00	1.00	1.00	1.00
Item 6- Removed garments after care with measures taken to avoid contamination [...]	1.00	1.00	1.00	1.00
Item 7- Discarded all waste from care provided in recipients [...]	1.00	1.00	1.00	1.00
**Item/Dimension3: Care**				
Item 8- Assessed vital signs:() RR= ; () SpO2 AA. Annotate: ; () Temperature= ; () HR=[...]	1.00	1.00	1.00	1.00
Item 9- Assessed breathing pattern: () Eupnea; () Dyspnoea [...]	1.00	1.00	1.00	1.00
Item 10- Assessed criteria of severe acute respiratory syndrome (SARS) [...]	1.00	1.00	1.00	1.00
Item 11- Collected sample for investigation of COVID-19 and sent it to [...]	1.00	1.00	1.00	1.00
Item 12- Investigated patient’s vaccinal situation for COVID-19: [...]	1.00	1.00	1.00	1.00
Item 13- Performed Notification of SARS and communicated with municipal[...]	1.00	1.00	1.00	1.00
Item 14- Initiated oxygen supplementation, if patient had SpO_2_ ≤ 94% AA [...]	1.00	1.00	1.00	1.00
Item 15- Not placed water or saline solution in humidifier when O_2_ supplemented [...]	1.00	1.00	1.00	1.00
Item 16- Used surgical mask on patients using nasal tube. [...]	1.00	1.00	1.00	1.00
Item 17- Observed level of consciousness, drop inSpO_2_and/or signs of respiratory distress[...]	1.00	1.00	1.00	1.00
Item 18- Verified whether patient presents criteria for indication for orotracheal intubation [...]	1.00	1.00	1.00	1.00
Item 19- Set up and tested functioning of mechanical ventilator [...]	1.00	1.00	1.00	1.00
Item 20- Coupled HEPA (High Efficiency Particulate Arrestance) filter to orotracheal tube [...]	1.00	1.00	1.00	1.00
Item 21- Sealed orotracheal tube with Reynalds forceps or plastic cap [...]	1.00	1.00	1.00	1.00
Item 22- Prepared suction device (aspirator) for closed system [...]	1.00	1.00	1.00	1.00
Item 23- Performed pre-oxygenation for 5 minutes using oxygen flow of [...]	1.00	1.00	1.00	1.00
Item 24- Assisted in intubation performed by physician and then confirmed [...]	1.00	1.00	1.00	1.00
Item 25- In the case of two failed orotracheal intubation attempts, assisted in [...]	1.00	0.88	0.88	1.00
Item 26- Performed clamping of orotracheal tube with Reynalds forceps [...]	1.00	1.00	1.00	1.00
Item 27- Performed aspiration of orotracheal tube and upper airways [...]	1.00	1.00	1.00	1.00
Item 28- Performed arterial gas exchange reading after 30 minutes of orotracheal intubation [...]	1.00	1.00	1.00	1.00
Item 29- Maintained pre-oxygenation non-re-inhalation face mask with O_2_ flow of [...]	1.00	1.00	1.00	1.00
Item 30- Not used manual resuscitator due to high risk of generation [...]	0.88	1.00	1.00	1.00
Item 31- In patients on mechanical ventilation, connection to closed circuit ventilator [...]	1.00	1.00	1.00	1.00
Item 32- Patient in prone position without advanced airway was quickly[...]	1.00	1.00	1.00	1.00
Item 33- Patient in prone position with invasive ventilation, chest compressions were [...]	1.00	1.00	1.00	1.00
Item 34- For defibrillation of patient in prone position, sternal paddle was placed in [...]	1.00	1.00	1.00	1.00
Item 35- Placed patient in bed and maintained upper portion of bed elevated between 30° [...]	1.00	1.00	1.00	1.00
Item 36- Positioned patients with RR > 24 bpm and hypoxemia with no signs [...]	1.00	1.00	1.00	1.00
Item 37- Positioned and/or assisted in positioning patient in prone position, [...]	1.00	1.00	1.00	1.00
Item 38- Maintained self-pronated patients with spontaneous ventilation [...]	1.00	1.00	1.00	1.00
Item 39- Informed team of suspicion or confirmation of COVID-19 [...]	1.00	1.00	1.00	1.00
Item 40- Transported patient with surgical mask in wheelchair [...]	1.00	1.00	1.00	1.00
Item 41- Made telephone contact with dispatcher of mobile urgent care service [...]	1.00	1.00	1.00	1.00
Item 42- Prepared body at site of occurrence of death [...]	1.00	1.00	1.00	1.00
Item 43- Wrapped body in three layers: roll body in sheets; place [...]	1.00	1.00	1.00	1.00
Item 44- Labeled outer transport bag with information related to biological risk [...]	1.00	1.00	1.00	1.00

Note: C= clarity; R= relevance; P= pertinence; Cp = comprehensiveness.

**Table 4:** *Checklist* for nursing care management of patients with suspicion or confirmation of infection by COVID-19 at 24-h UCU.

Nurse:		Patient:			
**Date:**		**Bed/Sector:**		Y	N
***Dimension 1: Management***					
1.Promoted distancing between patients and minimized circulation of people and patients with respiratory symptoms.					
2.Prioritized radiographic exam so that patient does not wait in common areas (reception, waiting room, corridor).					
3.Minimized patient’s contact with other patients during their stay in emergency room or ward and observation room, closed curtains and increased distancing between beds when possible.					
4.Maintained small team, at most, three professionals during orotracheal intubation.					
***Dimension2: Biosafety***					
5.Put on garments prior to onset of patient care in anteroom of the emergency room or isolation room. Necessary equipment and way of performing procedure were in accordance with risk of exposure:					
Droplet and contact precaution, in the following sequence: 1- hand sanitization; 2- Long-sleeve apron or gown, knit or elastic wrist cuffs (minimum grammage: 30g/m^2^); 3- surgical mask; 4- protective eyewear; 5- cap; 6- gloves.					
Aerosol precaution, in following sequence: 1- hand sanitization; 2- Long-sleeve waterproof apron, knit or elastic wrist cuffs and open back (waterproof structure and minimum grammage of 50 g/m^2^); 3- N95 or PFF2 mask; 4- protective eyewear or face shield; 5- cap; 6- gloves.					
6.Removed garments after care with measures taken to avoid contamination, using following removal sequence: In patient room or emergency room: 1- remove gloves; 2- remove apron; 3- sanitize hands; leave room or emergency room where patient is located: 4- sanitize hands; 5- remove cap; 6- remove protective eyewear or face shield; 7- sanitize hands; 8- remove N95 or PFF2 mask; 9- sanitize hands.					
7.Discarded all waste from care provided in recipients for infectant waste, which will subsequently be labelled COVID-19.					
***Dimension3: Care***					
Initial patient care	8.Assessed vital signs: ( ) RR = ___ ( ) SpO_2_ AA Annotate: ___( ) Temperature = ___ ( ) HR = ___ _________				
	9.Assessed breathing pattern: ( )Eupnea ( ) Dyspnoea ( ) Tachypnoea ( ) Bradypnea ( )Signs of respiratory effort.( )Use of accessory muscles.( )Signs of cyanosis.				
Criteria for definition, reporting and conduct in SARS	10.Assessed criteria of severe acute respiratory syndrome (SARS) in patients with influenza syndrome: ( ) Dyspnoea *OR* ( ) Respiratory distress *OR* ( ) Persistent pressure in chest *OR* ( ) SpO_2_< 95% in ambient air *OR* ( ) Cyanosis in lips or face				
	11.Collected sample for investigation of COVID-19 and sent it to reference laboratory (Adolfo Lutz Institute), in accordance with local flow: ( )Nasal oropharyngeal swab (rapid antigen test) – SARS: 1st to 7th day of symptom; ( ) Nasal oropharyngeal swab (RT-PCR) – SARS: 1st to 14th day of symptom; ( )Blood sample (rapid IgM/IgG test) – SARS: beginning with 8^th^ day of symptom (NOTE: not recommended for diagnosis of vaccinated patients due to expected vaccinal response)				
	12.Investigated patient’s vaccinal situation for COVID-19: Laboratory/manufacturer of vaccine:_____; Number of doses received:_____.				
	13.Performed Notification of SARS and communicated with municipal epidemiological surveillance, in accordance with local flow.				
***Respiratory care for patients with suspicion or confirmation of COVID-19***					
Recommendations for ventilatory care: non-invasive	14.Initiated oxygen supplementation, if patient had SpO_2_ ≤ 94% AA *OR*RR ≥ 24 bpm. -Offered oxygen through nasal tube 1 to 6 L/min. Annotate (L/min): __________				
	- Offered oxygen through mask with non-re-inhalation reservoir 6 to 15 L/min. Annotate (L/min): __________				
	15.Not placed water or saline solution in humidifier when O_2_supplemented.				
	16.Used surgical mask on patients using nasal tube.				
	17.Observed level of consciousness, drop inSpO_2_and/or signs of respiratory distress in patient with high oxygen flow (>10 L/min)				
Recommendations for ventilatory care: invasive	18.Verified whether patient presents criteria for indication for orotracheal intubation and communicated to medical team, in accordance with following criteria: -Respiratory failure and lowered level of consciousness and/or increase in ventilatory work/high ventilatory effort; need for O_2_supplementation > 5L/m into maintain SpO_2_>94% orRR ≤ 24 bpm; retention of CO_2_ (PaCO_2_ > 50mmHg and/or pH< 7.25).				
	19.Set up and tested functioning of mechanical ventilator, adjusting it with initial parameters of mechanical ventilation after orotracheal intubation in accordance with medical orientation, respecting the following recommendations: ❖ Modality: controlled volume; tidal volume: 6 ml/kg of predicted weight; FiO_2_ 100%; initial PEEP of 10 cmH_2_0; RR: 24/min (20 to 28/min); inspiratory flow: 60 L/min (40-80 L/min) or I:E ratio of 1:2 to 1:4.				
	20.Coupled HEPA (*High Efficiency Particulate Arrestance*) or HMEF (*Heat and Moisture Exchanger*)filter to orotracheal tube and corrugated extension.				
	21.Sealed orotracheal tube with Reynalds forceps or plastic cap (or rubber stopper) when used guide wire or Bougie for intubation.				
	22.Prepared suction device (aspirator) for closed system.				
	23.Performed pre-oxygenation for 5 minutes using oxygen flow of 15 L/min with care to avoid leakage *or* use non-re-inhalation mask.				
	24.Assisted in intubation performed by physician and then confirmed position of tube by capnography (preferably).				
	25.In the case of two failed orotracheal intubation attempts, assisted in passage of extra-glottal device (laryngeal mask) coupled to a HEPA or HME filter.				
	26.Performed clamping of orotracheal tube with Reynalds forceps when its disconnection or the exchange of the circuit of the mechanical ventilator is necessary.				
	27.Performed aspiration of orotracheal tube and upper airways using closed suction system, whenever necessary.				
	28.Performed arterial gas exchange reading after 30 minutes of orotracheal intubation and when requested by the medical team.				
***Recommendations for cardiorespiratory arrest***					
Care for cardiorespiratory arrest	29.Maintained pre-oxygenation non-re-inhalation face mask with O_2_flow of 6-15 L/min until intubation to avoid generation of aerosols.				
	30.Not used manual resuscitator due to high risk of generation of aerosols and contamination of team.				
	31.In patients on mechanical ventilation, connection to closed circuit ventilator was maintained and set under medical orientation to “RCP/cardiorespiratory arrest” function of equipment; equipment without this function was adjusted to non-asynchronous ventilation with the following recommendations: ventilatory mode: asynchronous; 100%FiO_2_; RR: 10 to 12; PEEP = 0.				
	32.Patient in prone position without advanced airway was quickly positioned in supine position and cardiopulmonary resuscitation manoeuvres initiated.				
	33.Patient in prone position with invasive ventilation, chest compressions were performed with hands in normal position over vertebrae T7 to 10.				
	34.For defibrillation of patient in prone position, sternal paddle was placed in dorsal region and apical paddles was placed on patient’s left side.				
***Nursing conduct in positioning and internal transport of patients***					
Positioning of patient	35.Placed patient in bed and maintained upper portion of bed elevated between 30° and 45° (semi-reclined or Fowler position) as tolerated by patient.				
	36.Positioned patients with RR >24 bpm and hypoxemia with no signs of respiratory failure in prone position or conscious pronation.				
	37.Positioned and/or assisted in positioning patient in prone position and repositioned devices, wires and catheters.				
	38.Maintained self-pronated patients with spontaneous ventilation under constant surveillance.				
Internal transport of patient	39.Informed team of suspicion or confirmation of COVID-19.				
	40.Transported patient with surgical mask in wheelchair or stretcher using devices to maintain oxygen support during transport.				
	***Nursing conduct for probable outcomes***				
Transfer to reference hospital	41.Made telephone contact with dispatcher of mobile urgent care service to request transport of patient to reference hospital units after confirmation of availability by CROSS (service regulation center).				
After death	42.Prepared body at site of occurrence of death.				
	43.Wrapped body in three layers: roll body in sheets; place body in waterproof bag; place body in second (outer) bag and disinfect with 70% alcohol or other sanitizing agent compatible with material of bag.				
	44.Labeled outer transport bag with information related to biological risk: COVID-19- biological agent – risk class 3; and then send to morgue.				

## Data Availability

The data that support the findings of this study are available on request from the corresponding author. The data are not public available due to restrictions, such as their containing information that compromise the privacy of research participants. That all listed authors meet the authorship criteria and that all authors agree with the content of the manuscript.
